# Investigating the mortality trend of gastrointestinal cancers in Babol, North Iran (2013–2021)

**DOI:** 10.1186/s12876-024-03189-9

**Published:** 2024-03-06

**Authors:** Pouyan Ebrahimi, Mohsen Karami, Sahar Delavari, Layla Shojaie, Seyed-Hossein Hosseini-Berneti, Fatemeh Bayani, Mehdi Moghaddasi, Ozra Babazade, Hossein-Ali Nikbakht

**Affiliations:** 1 Student Research Committee, Babol University of Medical Science, Babol, Iran; 2 Infectious Diseases and Tropical Medicine Research Center, Health Research Institute, Department of Parasitology and Mycology, Babol University of Medical Sciences, Babol, Iran; 3 Institute for the Developing Mind, Children’s Hospital Los Angeles, , Keck School of Medicine, University of Southern California, Los Angeles, CA USA; 4 Division of GI/Liver, Department of Medicine, Keck school of Medicine, University of Southern California, Los Angeles, CA USA; 5 Department of Health, Health Systems Research, Health Research Institute, Babol University of Medical Sciences, Babol, Iran; 6 Social Determinants of Health Research Center, Health Research Institute, Department of Biostatistics & Epidemiology, School of Public Health, Babol University of Medical Sciences, Babol, Iran

**Keywords:** Gastrointestinal cancers, Trends, Mortality, Iran

## Abstract

**Background and aim:**

This study aims to examine the mortality rate and trend of gastrointestinal cancers, particularly gastric cancer, as the leading cause of death among cancers in northern Iran over a 9-year period. In light of the changing incidence and mortality rates of cancer in Iran and around the world, the importance of these diseases in people’s lives, and the necessity of updating and monitoring the trend of cancer mortality, we have decided to report on the mortality trend of gastrointestinal cancers, based on crude and age-standardized rates.

**Method:**

This study is a cross-sectional examination of deaths caused by gastrointestinal cancers in Babol city, Iran, between 2013 and 2021. Data was collected from the cause of death registration and classification system of Babol University of Medical Sciences. Population estimation was obtained from the latest census reports. The crude and age-standardized mortality rates and trends of the cancers were calculated.

**Results:**

Overall, there were 1345 deaths from gastrointestinal cancers with an average age of 69.11 ± 14.25 years. The crude and age-standardized rates of these cancers rose from 24.1 to 20.1 per hundred thousand people in 2012 to 29.5 and 25.5 per hundred thousand people, respectively. This trend became more prevalent significantly with the increase of each decade of age for both men (P-value Trend = 0.002) and women (P-value Trend = 0.012). An analysis of gastrointestinal cancers revealed a decreasing trend for cancers of the small intestine, an increasing trend for cancers of the colon, pancreas, and gallbladder, and a stable trend for the remaining cancers over the study period.

**Conclusion:**

The age-standardized rate and the number of gastrointestinal cancers is rising, highlighting the importance of preventative measures such as screening, increasing public awareness, and appropriate diagnostic methods.

## Introduction

The formation of tumors results from cells in certain body areas growing and multiplying excessively. Cancer may develop when these tumors become malignant [1]. Cancers have been introduced as the first or second cause of death in many countries in 2020 [2]. In 2020, the World Health Organization’s International Agency for Research on Cancer reported over 19 million new cases of cancer and almost 10 million deaths worldwide, affecting individuals of all ages and genders. Although men had a higher mortality and incidence rate, the five-year prevalence of cancer was higher in women. According to WHO’s classification, the Eastern Mediterranean Region had the lowest incidence and mortality rate of cancer among all regions, with Iran ranked 27th in terms of both incidence (131,191 per 100,000 individuals) and death (79,136 per 100,000 individuals) of cancer, in all ages and genders [3]. The rise in risk factors for gastrointestinal cancers, such as infection with Helicobacter pylori, urbanization, and lifestyle, has led to gastrointestinal cancers, particularly esophageal, gastric, and colorectal cancers, becoming increasingly significant globally. These cancers now play a major role in the global burden of cancer [2, 4].

In Iran, gastrointestinal cancers, including gastric, colorectal, liver, pancreas, and esophagus, account for 49.8% of the ten most deadly cancers [3]. The symptoms of these cancers can vary from being asymptomatic to exhibiting symptoms such as swallowing difficulties, abdominal pain, and bleeding. These symptoms vary depending on the specific location of the cancer within the digestive system [5]. Diagnosis of these cancers depends on the affected area and can include methods such as endoscopy, colonoscopy, and Endoscopic Retrograde Cholangiopancreatography (ERCP) [6, 7]. Treatment for these cancers is determined based on the patient’s condition and the stage of the disease and can include medication, chemotherapy, surgery, and other alternative therapies [8, 9].

It is essential for the social, economic, and health policies of every country to aim toward increasing life expectancy and reducing mortality rates [10]. Additionally, tracking mortality trends helps the healthcare system to set appropriate public health priorities and allocate resources accordingly. Monitoring and evaluating the effectiveness of preventive and therapeutic measures can also be improved by analyzing mortality data [11]. These data serve as indicators for the causes of diseases in epidemiological studies. In Iran, the Civil Registration and Vital Statistics (CRVS) system is currently in place in 30 provinces and records and categorizes deaths based on their causes. It is the best source of data for estimating cancer mortality [12].

Babol, located in northern Iran, and is the second-largest city in the region with a population of over 500,000 and an area of approximately 15,781 square kilometers [13]. Considering the changes in the incidence and mortality of cancers in Iran and the world, the significance of these diseases in people’s lives, and the need to monitor the trend of cancer mortality, we have decided to present the trend of mortality, specific and standardized rates. We will also compare the results with national and global statistics over 9 years and examine the epidemiological characteristics of gastrointestinal cancers in Babol city.

## Methods

This cross-sectional study focused on all recorded deaths from gastrointestinal cancers in Babol city between 2013 and 2021. It was conducted using the cause of death registration and classification system of Babol University of Medical Sciences and was approved by the Ethics Committee of Babol University of Medical Sciences with the code MUBABOL.HRI.REC.1401.153. The information on causes of death was collected from the death registration and classification system maintained by the Health Vice-Chancellor of the University of Medical Sciences. The system employed in this study relies on credible sources, including funeral homes, forensic medicine, hospitals, and physicians experienced in registering causes of death.

Initially, deaths caused by gastrointestinal cancers were analyzed qualitatively. Furthermore, various quality checks were performed to ensure the accuracy of the data. Involving identifying and correcting duplicated cases, verifying the consistency of the data with other recorded information, examining the causes of death for implausibility based on factors such as sex and age, and eliminating causes of death in ill-defined cases. The causes of death were reviewed and corrected by experts when necessary, using information records. After revisions, the ill-defined data were redistributed, and once approved by the program officials in the Ministry of Health, they were used and reported in this study.

The causes of death were categorized using the 11th edition of the International Classification of Diseases and Mortality (ICD-11). The codes for gastrointestinal cancers included: C15 (esophagus), C16 (stomach), C17 (small intestine), C18 (colon), C19 (rectosigmoid junction), C20 (rectum), C21 (anus and anal canal), C22 (liver and intrahepatic bile duct), C23-24 (gallbladder), C25 (pancreas), and C26 (malignant neoplasms of other and obscure parts of the digestive organs) [14]. Oral cancer was not included in this study as it falls under the category of “oral cavity and lip cancers”.

The sampling method employed in this study was determined by the recorded cases of deaths caused by gastrointestinal cancers during the specified period. To calculate and compare the mortality rate, an estimate of the population was created for each year, considering population growth between census years. The population estimation for different age groups was determined using the difference between the age groups recorded in the census years and the overall population for the years 2013 to 2021.

The study reported the mean and standard deviation for quantitative variables and frequency and percentage for qualitative variables. The crude mortality and age-standardized rate (ASR) with a 95% confidence interval were also reported. The city’s population was obtained from the Iranian Statistics Center census to calculate the crude mortality rate. To calculate the age-specific standardized rate (ASR), the IARC global standard population was used along with the Globocan standard population and the direct method for 100,000 people. The trend in mortality between the specified years was analyzed based on the Chocran-Armitage-Trend Test. The analysis was conducted using STATA version 14 software with a significance level of *P* < 0.05. In addition, we created our figures using Excel 2016.

## Results

This study found that 1345 cases died from gastrointestinal cancers in Babol during 9 years from 2013 to 2021. Of these cases, 832 (61.9%) were male, and over half of the deaths, 710 (52.8%), were among urban residents. Regarding the cause of cancer, gastric cancer was the leading cause of death, with 538 (40.0%) cases. The other leading causes of death were: 249 colon cancer (18.5%), 168 (12.5%) liver and intrahepatic bile duct cancers, esophagus cancer 110 (8.2%), pancreas cancer 105 (7.8%), other parts of digestive organs 88 (6.5%), small intestine cancer 35 (2.6%), rectum cancer 20 (1.5%), from gallbladder cancer 17 (1.1%), cancer of the rectosigmoid junction 9 (0.7%), and cancer of the anus and anal ducts 6 (0.4%).

**Fig. 1:**
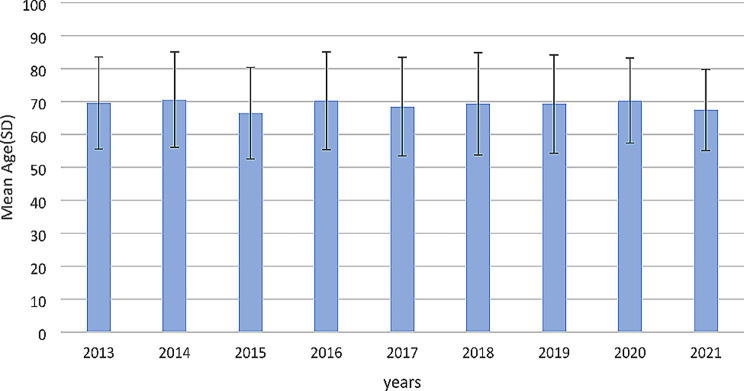
Average (standard deviation) age of gastrointestinal cancers in the studied years

**Fig. 2:**
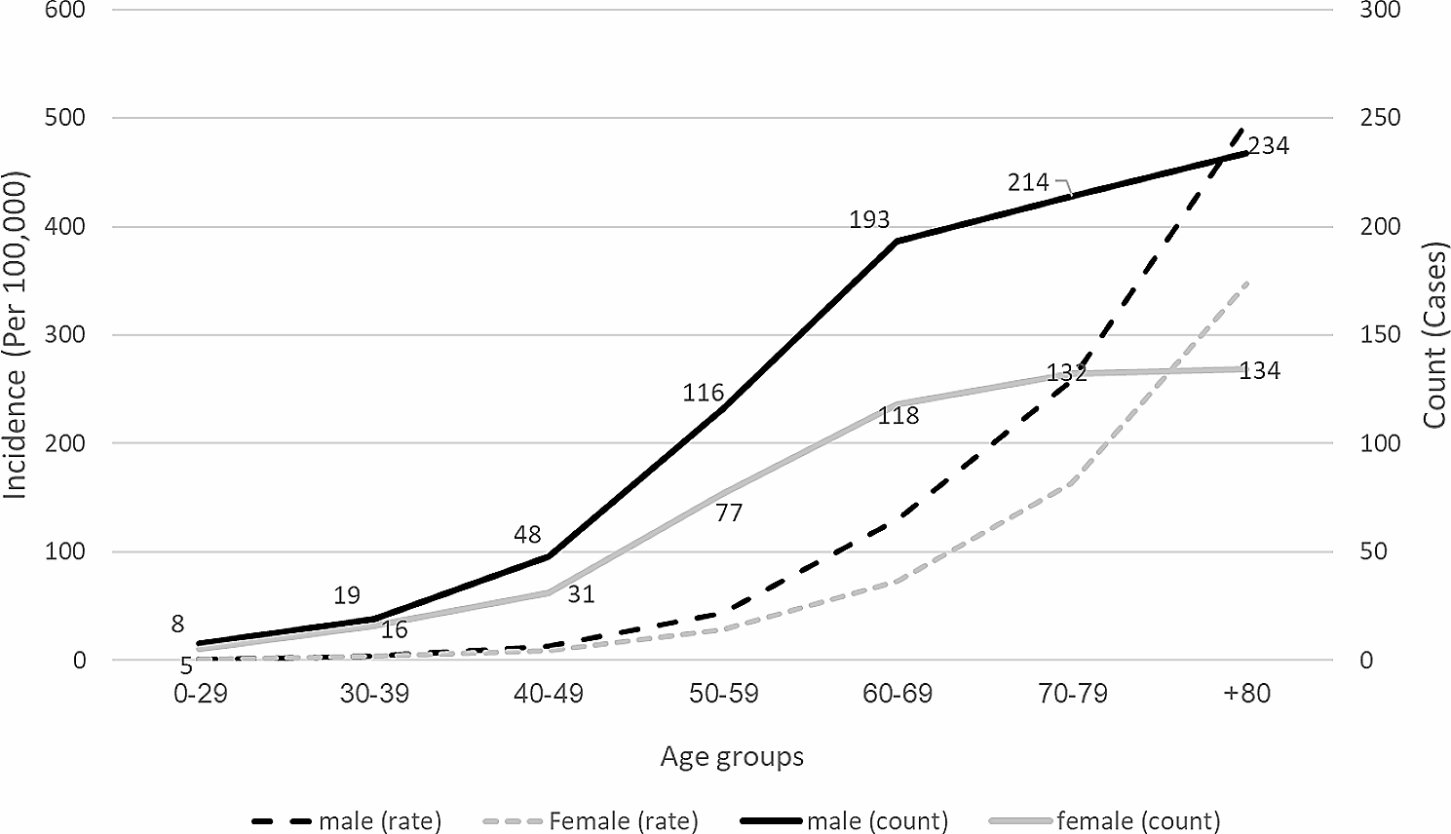
Examining the trend of the mortality rate and the rate of death per hundred thousand population due to gastrointestinal cancers according to age groups in Babol, Iran (2013–2021) (P-value Trend = 0.002 for men and P-value Trend = 0.012 for Women)

The mean and standard deviation of the patient’s overall age was 69.1 ± 14.3 years, ranging from 11 to 100 years old. The average age of men was 69.4 ± 14.1 years, while the average age for women was 68.7 ± 14.5 years. However, this difference between genders was not statistically significant (*P* = 0.400). The average age of death among gastrointestinal cancers was highest in the following order: gallbladder cancer (73.1 ± 10.3 years), esophagus (72.6 ± 12.4 years), and gastric cancer (70.2 ± 13.6 years). On the other hand, the cancers with the lowest average age of death are rectal cancer (59.0 ± 15.9 years), anus and anal canal cancer (61.8 ± 8.7 years), and rectosigmoid junction cancer (15.9 ± 1/63 years). The average and standard deviation of the age of death cases per year are also displayed in Fig. 1. An analysis of deaths by age group revealed that the number of deaths in both genders consistently increased, which was statistically significant (*P* < 0.05), as well as the overall mortality rate per a hundred thousand population (Fig. 2).

**Fig. 3:**
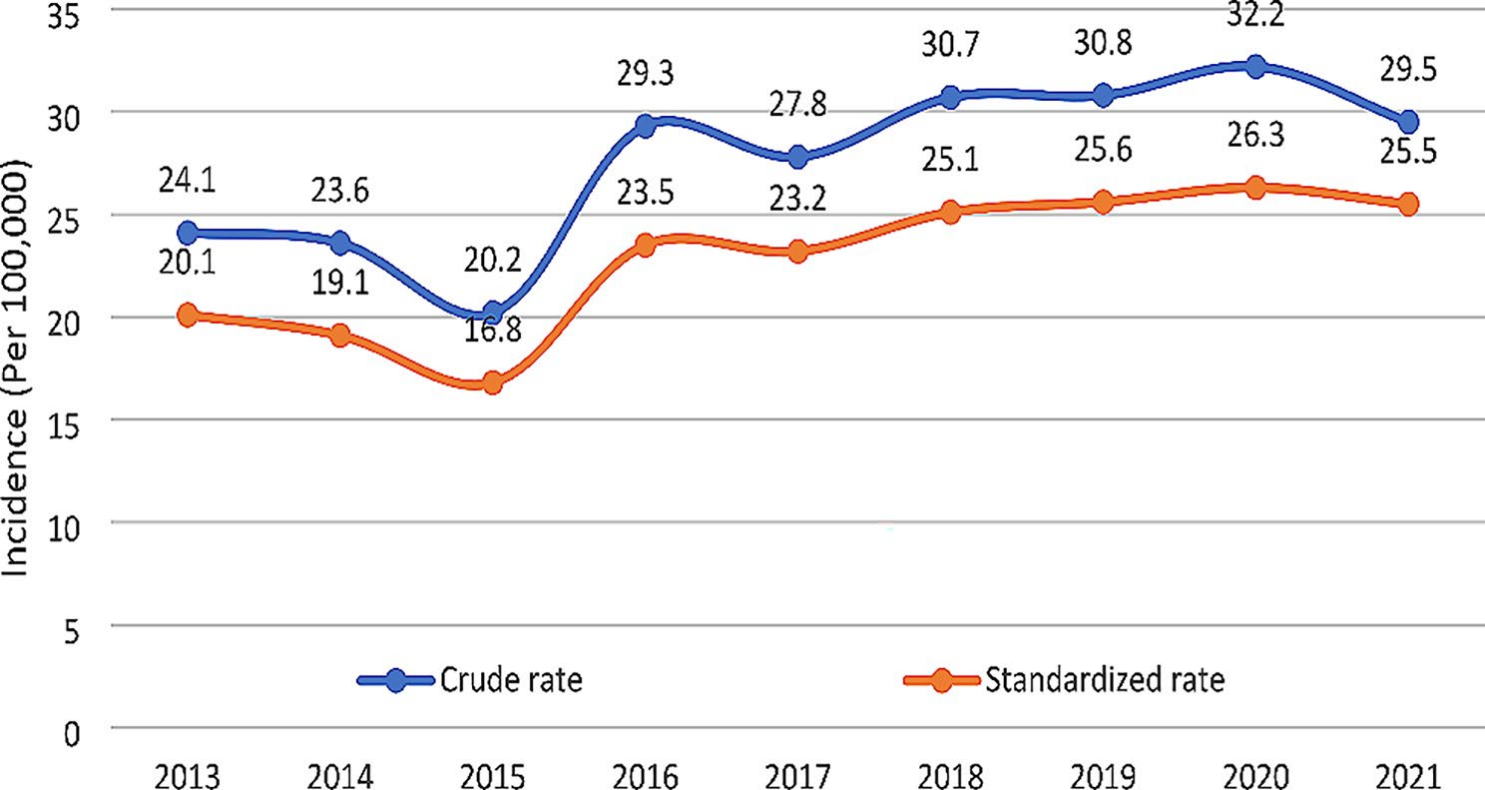
Crude and age-standardized mortality rates of gastrointestinal neoplasms per 100,000 population in Babol (Iran) during the years 2013–2021

The crude mortality rate was higher than the age-standardized mortality rate (ASMR) for gastrointestinal cancers in all cases. The mortality rates for gastrointestinal cancers in 2012 were recorded as 24.1 (crude rate) and 20.1 (age-standardized rate) per 100,000 people, but these rates increased to 29.5 and 25.5 per 100,000 people, respectively, by 2021. This increasing trend in mortality rate was found to be statistically significant (*P* < 0.001) (Fig. 3). The mortality rate of gastrointestinal cancers was also analyzed based on gender and year. The results indicated that the first three years of the study had the lowest crude and age-standardized mortality rates in women, which was less than one-third of patients per hundred thousand people. The highest frequency, almost one-third of patients per hundred thousand people, was recorded in the last three years of the study: 2018, 2019, and 2021 (Table 1).

The mortality rate of gastrointestinal cancer showed a significant increase in the standardized rates for cancers of the gallbladder, rectosigmoid junction, colon, and pancreas. Small intestine cancer was the only cancer type with a significant decrease during this period. There was no significant change in the mortality rates of the esophagus, gastric, rectum, anus, anal canal, liver, and intrahepatic bile duct cancers, except for gastric cancer, which had a slight increase. The highest crude and standardized rates were observed in gastric, colon, and liver cancers. (Table 2).

**Table 1:** Crude and age-standardized mortality rate of gastrointestinal cancer according to the studied years and gender per hundred thousand population in Babol (Iran) 2013–2022

Years	Male			Female		
	Crude death rate	Age- Standardized mortality rate		Crude death rate	Age- Standardized mortality rate	
		rate	95% Confidence Interval		rate	95% Confidence Interval
2013	32.3	26.6	20.6–32.5	15.8	13.4	9.2–17.6
2014	27.7	22.1	16.8–27.5	19.5	16.0	11.5–20.6
2015	23.1	19.2	14.3–24.2	17.3	14.4	10.1–18.6
2016	36.2	17.8	23.2–35.4	22.3	29.3	13.1–22.4
2017	23.1	19.4	14.5–24.3	32.4	26.9	21.1–32.8
2018	39.6	31.6	25.4–37.8	21.7	18.4	13.6–23.2
2019	38.7	33.0	26.5–39.5	22.9	18.6	13.9–23.3
2020	38.5	31.2	25.1–37.3	25.8	21.3	16.3–26.3
2021	36.6	32.5	26.1–38.9	22.2	18.8	14.1–23.5

**Table 2:** The trend of crude and standardized mortality rates by the studied years for each of the gastrointestinal cancers per one hundred thousand population in Babol (Iran) 2013–2022

Years	Rate	Esophagus	Stomach	Small Intestine	Colon	Rectosigmoid Junction	Rectum	Anus and Anal Canal	Liver and Intrahepatic Bile Ducts	Gallbladder	Pancreas	Other and Ill-defined Digestive Organs
2013	Crude	2.7	9.2	2.2	3.1	0	0.2	0.2	6.3	0	0.2	0
	Standardized	2.6	7.6	1.8	2.6	0	0.2	0.2	5.1	0	0.1	0
2014	Crude	2.3	11.0	2.7	2.1	0	0.2	0	4.6	0	0.2	0.4
	Standardized	1.8	9.0	2.1	1.5	0	0.2	0	4.0	0	0.2	0.3
2015	Crude	2.5	8.8	0.2	2.9	0	1.3	0.2	1.5	0.2	2.1	0.6
	Standardized	1.8	7.5	0.1	2.5	0	1.2	0.2	1.3	0.2	1.7	0.4
2016	Crude	2.8	11.8	0.4	3.8	0.2	1.3	0	2.8	0	3.9	2.3
	Standardized	2.1	9.7	0.3	3.1	0.1	1.1	0	2.2	0	3.3	1.5
2017	Crude	2.4	11.1	0.4	6.3	0	0	0	2.4	0.2	2.2	2.8
	Standardized	1.9	9.3	0.3	5.2	0	0	0	2.1	0.2	1.7	2.5
2018	Crude	1.8	6.4	0.4	7.5	0.4	0.2	0.2	2.7	0.5	3.3	3.8
	Standardized	1.6	5.1	0.3	5.9	0.2	0.1	0.1	2.3	0.4	2.9	3.1
2019	Crude	1.3	14.1	0	6.3	0.4	0	0.4	2.9	0.5	2.7	2.3
	Standardized	1.1	11.0	0	5.4	0.3	0	0.3	2.4	0.4	2.7	1.8
2020	Crude	3.4	12.6	0.2	7.5	0.4	0	0	4.8	0.5	1.8	1.1
	Standardized	2.7	10.1	0.2	5.9	0.4	0	0	4.0	0.5	1.6	1.0
2021	Crude	1.2	10.9	0.4	6.1	0.4	0.5	0.2	3.2	1.1	2.8	2.8
	Standardized	0.9	9.2	0.3	5.5	0.3	0.5	0.2	2.6	0.9	2.7	2.4
P-trend		0.206	0.074	< 0.001	< 0.001	0.019	0.144	0.699	0.163	< 0.001	0.002	< 0.001

## Discussion

In our study, we observed a significant increase in the gastrointestinal cancer mortality rate in the north of Iran over 9 years (2013–2021). Gastric cancer was the most common type, followed by colon and liver cancers. The average age of cancer patients was constant, but the mortality rate increased with age. Men had a higher mortality rate from gastrointestinal cancers than women (1.62:1 ratio).

The study showed that the standardized age rate for gastrointestinal cancers in the north of Iran increased from 20.1 to 25.5 per 100,000 people between 2013 and 2021, a significant increase of 1.3 times. Gastrointestinal cancers in men were increasing, while in women, it remained constant. In another study conducted in the center of Iran during the years 2005–2015, there was an increase in the age-standardized rate of gastrointestinal cancers in both sexes, from 15.0 to 19.4 per 100,000 people between 2005 and 2015 [15].

Another study using data from Iran’s death registry for 2001–2015 reported a decreasing trend in gastrointestinal cancers for both men and women. The trend was faster in men, but the incidence of gastrointestinal cancers was still higher in men than in women [16]. Our study’s findings is consistent with the increasing trend observed in other studies. However, there are notable differences in predicting the trend for women, and one potential explanation for these differences could be related to the age limit used in the other studies. Another factor to consider is the implementation of a systematization and online death registration system in Iran in 2014–2015, which might have led to a more detailed examination of the registered data from that period.

A study conducted in Iraq during the years 2000–2016 reported an increasing trend in gastrointestinal cancers [17]. In another study conducted in China during 2010–2014, a decreasing trend in major gastrointestinal cancers was observed [18], and this trend has continued for most gastrointestinal cancers in China in recent years [19]. The differences in trends observed in different countries and regions can be attributed to factors such as the higher prevalence of late-stage disease, late diagnosis, and risk factors in the region. The difference can also be due to the different years of the studies and the data collection methods used. Other factors that could contribute to the difference include the presence of other risk factors such as the consumption of hot tea, higher cigarette use, and higher prevalence of Helicobacter pylori in the north country.

Our study found that the mortality rate increased with age. A study conducted using GLOBOCAN 2020 data found that gastrointestinal cancers were more prevalent in men than women in all three epidemiological regions (China, America, and Europe) and at all ages [4]. The reason for the higher incidence of gastrointestinal cancers in men compared to women is likely due to high-risk behaviors such as alcohol consumption and drug abuse, which are more prevalent in men compared to women. Women are exposed to other risk factors, such as inactivity, but this factor contributes to a higher incidence of other cancers rather than gastrointestinal cancers [20].

In our study, gastric cancer was the most common gastrointestinal cancer, with a standardized rate of 9.2 per hundred thousand people. It was responsible for two-fifths of all deaths due to gastrointestinal cancers. The prevalence of gastric cancer remained stable in the north of Iran over the 9-year study period. A review study conducted by Farhood et al. in 2018 found that gastric cancer is the leading cause of death from cancer in Iran and that its prevalence is increasing, particularly in northern provinces such as Mazandaran [21]. The mortality rate from gastrointestinal cancers in Babol city is higher compared to the global average of 7.7 deaths per 100,000 people [3]. This rate surpasses the rate in Asia, which is at 10.0 deaths per 100,000 people [3]. However, it is lower than in Iran, which stands at 15.5 deaths per hundred thousand people [3]. This disparity in mortality rates between our city and Iran might be due to the location of the patient sample, as the study was conducted only in the north of the country, where the trend of gastrointestinal cancers is prevalent. Additionally, environmental factors such as the high prevalence of Helicobacter pylori infection, smoking, gastric reflux, and high salt consumption are believed to play a significant role in the occurrence of this cancer in Iran [21]. The standardized rate of gastric cancer in China is 13.4 per hundred thousand people, according to a study by Li et al. in 2021 [22]. Some regions, such as East Asia and parts of Latin America, continue to experience a high burden of gastric cancer, with higher incidence and mortality rates than other regions [23].

In this study, colorectal cancer was the second most common gastrointestinal cancer, with an age-standardized rate of 6.5 cases per hundred thousand people. Its trend showed a slight increase in colon and rectosigmoid cancer and remained unchanged in other areas. A similar study conducted in central Iran over 11 years from 2005 to 2011 found that colorectal cancer had a rate of 3.4 cases per hundred thousand people and was responsible for 13.9% of deaths. This study showed a significant increase in the incidence of colon cancer and a constant trend in the incidence of rectosigmoid cancer [15]. The standardized rate (per hundred thousand people) of colorectal cancer in Iran (10.9) [3] was higher compared to Asia (8.6) [3] and the world (9.0) [3] and even our study. It is more likely that this can be attributed to the improvement in diagnostic methods and screenings for this cancer in the past years in the country, as well as the change in diet, especially the consumption of more processed foods. In the study conducted on the 1985–2016, the colon cancer death trend has been increasing in Eastern and Southern Europe, unlike most Asian countries, America and Canada. There has been a constant trend for rectal cancer in most regions of the world [24]. This variation in trends in different areas could also be attributed to different levels of exposure to known risk factors for colorectal cancer, such as low fruit and vegetable intake, high levels of obesity, a sedentary lifestyle, alcohol consumption, and a meat-rich diet.

In our study, liver cancer was found to have an age-standardized rate of 2.6 per hundred thousand people and accounted for 12.5% of the deaths from gastrointestinal cancers. There was no change in the mortality rate of this cancer during the study period. According to the latest statistics from Globucon 2020, the standardized rate of liver cancer in Iran is 6.4, in Asia, it is 10.7, and globally it is 8.7 per hundred thousand people [3]. The significance of liver cancer can be seen as it is the leading cause of death due to cancer in Asia. In comparison, Babol city has a lower standardized rate. However, the rate in central regions of Iran is reported to be 3.8, making it the second most common cause of cancer deaths after gastric cancer, with 22.9% of cancer deaths being attributed to it [24], which is higher than the rate reported in our study.

This disparity between the two regions could be attributed to the late diagnoses and more advanced cancer stages at the referral time. In Iran, the age-standardized rate of liver cancer has risen from 1.1 to 5.6 per 100,000 people between 1990 and 2015 [25]. This rising trend has been the subject of many of rhe recent studies [16]. Liver cancer mortality rate decreased globally between 1990 and 2019 due to proper treatment and preventive measures [26]. Steps to control risk factors, such as administering appropriate vaccinations for hepatitis B and C, managing type 2 diabetes through dietary and lifestyle changes, and reducing smoking, can help control the incidence of this cancer [2, 27].

## Limitations and strengths

A limitation of this study is the omission of variables such as employment and education status, which were not considered due to high rates of missing data. Additionally, the mortality rate of gastrointestinal cancers may be underreported due to improper diagnosis or limited records. On the other hand, the strengths of this study include the presentation of the standardized rate and trend of each type of gastrointestinal cancer over 9 years.

## Conclusion

This study concluded that the trend of gastrointestinal cancers in Babol city, Iran, has been increasing from 2013 to 2021 and is not limited to specific ages or genders. The study also highlights the need for better control and screening of various gastrointestinal cancers. The late presentation of symptoms and lack of appropriate diagnostic methods contribute to the late diagnosis of these cancers, making primary screening and public awareness important in reducing the risk of these cancers. Further studies are needed to determine the contribution of various risk factors in developing these cancers.

## Data Availability

The corresponding author’s dataset during the current study are available upon reasonable request.
